# Editorial: Ancient diseases and medical care: Paleopathological insights

**DOI:** 10.3389/fmed.2023.1140974

**Published:** 2023-02-03

**Authors:** Sahar Saleem, Raffaella Bianucci, Francesco M. Galassi, Andreas G. Nerlich

**Affiliations:** ^1^Department of Radiology, Kasr Al Ainy Faculty of Medicine, Cairo University, Cairo, Egypt; ^2^Department of Cultures and Societies, University of Palermo, Palermo, Italy; ^3^Legal Medicine Section, Department of Public Health and Pediatric Sciences, University of Turin, Turin, Italy; ^4^The Ronin Institute, Montclair, NJ, United States; ^5^Department of Archaeology, College of Humanities, Arts and Social Sciences, Flinders University, Adelaide, SA, Australia; ^6^Institute of Pathology, Academic Clinic Munich-Bogenhausen, Munich, Germany

**Keywords:** paleopathology, paleoradiology, paleohistology, stable isotope analysis, ancient diseases

Diseases are the oldest companions to humankind. They have always deeply influenced human evolution, human history and the biological development of human beings. Paleopathology is the scientific discipline that investigates ancient diseases and related conditions in skeletal and soft tissue remains from prehistory until the very recent past. The analysis of the direct sources (osteological remains and mummies) requires beyond a careful investigation also the integration of all available indirect sources such as contemporary written sources (if any), papyri, images, inscriptions, representations, statuettes, medical and non-medical texts, as suggested by early founders of the “Paleopathology Association” (PPA), including the Cockburns (1). Indirect sources provide additional information about socio-cultural practices and religious beliefs of ancient populations (the relationship between healthy and diseased individuals in past communities, the way diseases were perceived in antiquity, how they were cured, how disabilities were handled and care was administered etc.).

The main basis for paleopathological investigations is the understanding of disease in its modern context. Currently, retrospective diagnoses are achieved resorting to a broad spectrum of techniques coming from various medical subspecialities, but also from bio- and cultural anthropology, (bio-geo) chemistry, biophysics and several other disciplines (2).

Thanks to a growing number of archaeological investigations, a plethora of ancient human remains (fossils, skeletons and isolated bones, natural and embalmed mummies) as well as written records (e.g., papyri, ancient tablets, medical textbooks etc.) and historic representations (such as figures, images, wall paintings and statuettes) have been made available for scientific investigation. These materials and information are time capsules permitting paleopathologists to identify a great variety of diseases, to observe their natural course and to learn about ancient medical therapies including application of remedies and surgical procedures. Beyond this, they allow the reconstruction of human activity patterns, interpersonal interactions, family relationships up to the identification of artificial manipulations, accidents and, evidence of interpersonal violence (i.e., murder) (3).

Within the past decades, the enormous advancement of diagnostic medical techniques permitted the use of a growing set of informational sources; however, the critical use of those techniques and the interpretation of data require special training which frequently extends beyond routine clinical applications. In particular, paleoradiology with its significant technical improvements of data handling, but also other disciplines such as paleohistology, paleomicrobiology including paleoparasitology and paleogenetics have enriched the spectrum of information; beyond this, stable isotope analysis, radiocarbon dating, investigation of dental calculus has broadened the data set.

Every investigational approach on ancient remains is based on the state of preservation of a given specimen, which may range from excellent to extremely poor. Regardless from the state of conservation, paleopathologists have always to bear in mind that they do examine the *vestigia* of past individuals and that their analysis and, later, conservation have to be performed following ethical and juridical rules. Particularly, the latter may vary from country to country.

At the beginning of each investigation, a stepwise approach with non-destructive analyses, mainly radiological techniques, has to be preferred. Several studies frequently resorted and still resort to X-ray technology; this is primarily due to the fact that X-ray portable instruments can be transported directly onto the field. Modern studies tend to apply advanced CT-techniques which provide much more information. These may be performed by use of existing clinical units requiring transport of the study material; in particular occasions, special scanning units may alternatively be brought to the site of excavation/ storage of human material, mainly complete human mummies (e.g., the CT scanning of the mummy of King Tutankhamun in Thebes). The Cairo Egyptian Museum is the only museum that has its own CT scan machine.

Multi-detector CTs (MDCT)s provide enormous data sets and permit extensive data evaluation including e.g., virtual unwrapping of covered human bodies without damage to the body. Nevertheless, recognizing that there are differences of opinion in the field of mummy studies, beyond this, recovery of further study material may be necessary which, however, requires tissue sampling (especially for all types of paleomolecular studies) and thereby some destruction and consumption of tissue material. While minimal invasive approaches, i.e., by endoscopy and biopsy are clearly to be preferred, on some occasions more extensive destruction and large-scale sampling may be necessary (if permitted by the legislation of the country where the remains are stored). Finally, the will of the descendants (if any) has to be taken into account and respected (4).

Despite this, there exists broad scientific consent that all interventions—may these be at small or large scale—require a clear scientific justification by formulation of precise study protocols and aims. Finally, paleopathology may provide deep insight into aspects of social behavior of historic populations, e.g., the management of disease in terms of “medical treatment,” care of disabled individuals and procurement of the dead body in accord with the respective religious believes.

The topic of “Ancient disease and medical care” provides a collection of high quality scientific papers which add important information to the paleopathological literature. The series of seven contributions covers not only various regions of the world (Egypt, South America, Europe), different time periods (15th century BC, 3rd-−1st century BC, 10th to 13th century AD and 16th century AD, respectively) but also different scientific techniques (paleoradiology, paleomicrobiology, paleohistology and radiocarbon analyses).

In five papers of this series, paleoradiology is the central analytical technique. The series of three papers by Saleem and Hawass report on paleoradiological investigations of important rulers of the ancient Egyptian Empire, in particular on the unique findings of the Pharaohs Seqenenre-Taa-II and Amenhotep-I. These studies provide a deep insight into history and contribute to our understanding of distinct events of the ancient Egyptian Empire.

The mummy of Pharaoh Seqenenre-Taa-II shows that the king was killed: he presented with multiple injuries to the head with a defect morphology that correlates well with the weaponry of the that-time invading Hyksos warriors. Detailed investigations further suggest that the King might have been imprisoned before death since the *postmortem* position of his arms suggest that they had been tied together before the Pharaoh's death—most likely behind the body. The king obviously died distant from the funeral place and there is good evidence that he was mummified in a location properly equipped for *lege artis* embalming.

The second paleoradiological paper describes the virtual unwrapping of the Egyptian King Amenhotep-I. A unique insight into life and death of this Pharaoh, a ca. 35 years old man without overt pathology, was achieved. The investigation showed that the mummy was subjected to rude treatment by ancient grave robbers which resulted in several extensive defects. As shown by the study, these defects were, then, restored through re-embalming procedures.

Lastly, Saleem et al. presented a third paleoradiological CT-scanning study performed on the mummy of the “Golden Boy,” a fully wrapped late Ptolemaic mummy. The excellently prepared, artificially embalmed mummy of a juvenile male shows a gilded head mask, a pectoral cartonnage and a large number of amulets including a golden tongue amulet and others. There was no evidence for obvious pathology. This study further highlights the value of CT scanning including the option of 3D-printing, a technique which can be further used for Museum displays.

Begerock et al. resorted to MDCT and 3-D reconstruction to study three natural Pre-Columbian South American mummies dating to a period between ca. 900 and 1,300 AD. The scientific evaluation showed that one male individual, known as Marburg Man, was victim of homicide. The individual displayed a severe trauma to the skull and was stabbed to the chest with laceration of the aorta and consequent death. The second adult male individual, known as Delémont Man, was also victim of interpersonal violence; his mummified remains reveal recurrent and possibly lethal traumata to the skull. The adult female individual, known as Delémont Woman, does not show evidence of pathologies or intra-*vitam* violence. The series of disruptions radiologically identified are due to taphonomic alterations.

The study by Nerlich et al. on a 16th century aristocratic infant mummy from an Austrian family crypt showed a surprisingly well-preserved naturally mummified infant of ~1–1.5 years of age; the child suffered from rickets (evidence of “rachitic rosary” is present) not associated with bowing of long bones. Additionally, a thickened subcutaneous fat tissue layer suggested the presence of *adipositas*, which was confirmed by skin histology. The combination of both diseases suggests that, despite an excellent nutritional supply, the infant suffered from vitamin-D-deficiency due to lack of necessary sun-light exposure for vitamin D processing. Finally, CT-scans showed sequels of pneumonia which may represent the cause of death.

The final two contributions cover a further aspect of paleopathology, namely paleomicrobiology. In the mini review study by Boualam et al. the spread and therapy of malaria in Europe have been traced since antiquity. This paper further describes research pathways for a renewed understanding of malaria eradication in Europe.

Finally, Van Der Walt and Keddy reported on the interplay between tuberculosis and depression over the last century with a particular focus on the role of isoniazid therapy with its effects on both diseases and its limitations. This study opens the way into modern day medical aspects of therapy—from paleopathology to modern medicine.

In summary, the papers of this topic have significantly contributed to the advancement of paleopathology and several of its subspecialities. Since all contributions of the topic are freely available, they are easily accessible resulting in high visibility of the papers. The high number of views of all contributions, each with several thousands of views, underlines the overall interest in this Research Topic and the high quality of the papers. It is therefore highly desirable to set up a second round of contributions in a follow-up.

## Author contributions

SS, RB, FG, and AN: all writing and correction of the paper. All authors contributed to the article and approved the submitted version.
